# Diseases of the Brain Due to Diseases of the Ear

**Published:** 1894-01-20

**Authors:** 


### DISEASES OF THE BRAIN DUE TO DISEASES OF THE EAR.

We have already mentioned that suppurative meningitis is a common complication of disease of the middle ear. It is important to remember that meningitis is more common in children than thrombosis of the lateral sinus, which is nearly always met with in adults. Often in children the meningitis is so insidious and the symptoms are so latent that it is not suspected till towards the end of the case. From the therapeutic point of view infective suppurative meningitis due to middle ear disease is less interesting than the other two complications, for it is nearly always fatal.

Whenever cerebral abscess or infective thrombosis is even suspected, it is the physician's duty to at once call in the aid of the surgeon, who should always begin by exposing the mastoid antrum, either by means of a bur or a gouge. This cavity should be well cleaned out, and if the mastoid cells are involved, they also must be obliterated and their contents removed. If necessary, the middle ear should also be opened and cleaned out, care being taken to avoid the facial nerve. The chorda tympani is almost always destroyed, but the patient is unaware of the consequent defect in taste because it is unilateral. If, as we are supposing, intra-cranial mischief is suspected, the operation is continued to the exposure of the lateral sinus, which, if it is diseased, should be opened and obliterated, and stuffed with iodoform and boracic acid. If the infective thrombosis has been extensive, the internal jugular vein should be ligatured in two places below the thrombosis, and divided between the ligatures. If a subdural abscess or meningitis is considered likely, the roof of the middle ear may be removed and the pus drained away through the opening thus made. Lastly, by means of an exploring needle, the temporo-sphenoidal lobe and the cerebellum may be thoroughly explored to see if an abscess is present in either.

The differential diagnosis between meningitis, cerebral abscess, and thrombosis of the lateral sinus is an important matter. The following are the chief points to which, in any patient suffering from chronic disease of the ear, attention should be directed when attempting to diagnose correctly which of these is present. If the rigors continue for several days, the probability is that the case is one of infective thrombosis; if they only last for the first few days, cerebral abscess is most likely present, and the complete absence of rigors indicates meningitis. The temperature is very important in a differential diagnosis. In cases of cerebral abscess, it is nearly always normal or subnormal till the abscess is opened, when for a day or two it may rise to about 100 deg., after which it falls to normal. With meningitis the temperature is raised —it is usually between 101 deg. and 103 deg., but it may be much higher—if it fluctuates widely, the fluctuations, both in their duration and in the period at which they occur, are quite irregular. Should thrombosis be present the temperature is always high, often reaching 106 deg.; it fluctuates widely and regularly, both in duration and extent. The pulse is also a valuable aid in diagnosis. If infective thrombosis be present, it is rapid, small, regular, and thready. On the other hand, if meningitis or cerebral abscess are present it is slow, not particularly small, and it may be irregular, especially in the case of meningitis. The pulse is less distinctive in meningitis than in abscess, for in infective meningitis it is sometimes quick and small, in cases of abcess never. The respirations are for the most part like the pulse; thus in thrombosis they are usually regular and rapid, especially if the lungs are infected secondarily; in abscess they are slow and often irregular; and in meningitis the breathing is not very distinctive, for while, if the posterior fossa is implicated, the respirations may be slow and irregular; often they are quick and regular. The mental condition of the patient not unfrequently helps us very much. It is very characteristic of cerebral abscess that he should lie in bed in a dazed condition, all his mental processes apparently going on very slowly. Thus when he is asked a question he does not answer for some time, and is unaware that he has not answered it immediately; also he will drop asleep while he is being spoken to, and will even do the same in the middle of a sentence that he is speaking. It is remarkable how in some cases almost directly the abscess is opened the mental condition improves. In meningitis there is no such constant mental condition. Often the patient gradually sinks into a state of coma, but even then there is not the slow cerebration which is so symptomatic of cerebral abscess. On the other hand, patients with meningitis are often violently delirious, a state rarely or never met with in those suffering from cerebral abscess. In infective thrombosis the mental condition is indicative of the general infection, rather than of any local condition, consequently there is usually present that low muttering delirium so typical of the typhoid state. In all three diseases anorexia and cerebral vomiting are present. In abscess, however, it is usually absent after the first few days. Diarrhœa is considerable evidence in favour of thrombosis, for it indicates general infection unless it can be shown that it is due to pus, which having trickled out of the Eustachian tube has been unknowingly swallowed by the patient. Considerable wasting during the illness is almost proof that the patient is suffering from abscess. Convulsions are common in meningitis, very rare in abscess, and still rarer in infective thrombosis. If paralysis is present, it is a sure indication of meningitis, especially if irregularly distributed. The only exception to this statement is that if the paralysis is such as could be caused by distension of the temporo-sphenoidal lobe or of the lateral lobe of the cerebellum it may be due to abscess. Instances of paralysis caused by an abscess are the hemiplegia due to upward pressure towards the motor region, and the paralysis of the third nerve due to downward pressure on it, met with in large abscesses of the temporo-sphenoidal lobe. Optic neuritis, if very acute and rapidly advancing, is indicative of meningitis; often it is absent in both abscess and thrombosis and when present is in an early stage. Local signs in the neck are strongly indicative of thrombosis. Very often the differential diagnosis is impossible, because all three or any two conditions may co-exist.

